# Cytokine exposure mediates transcriptional activation of the orphan nuclear receptor Nur77 in hematopoietic cells

**DOI:** 10.1016/j.jbc.2021.101240

**Published:** 2021-09-24

**Authors:** Orsola di Martino, Haixia Niu, Gayla Hadwiger, Margaret A. Ferris, John S. Welch

**Affiliations:** 1 Department of Internal Medicine, Washington University, St Louis, Missouri, USA; 2 Division of Experimental Hematology and Cancer Biology, Cancer & Blood Diseases Institute, Cincinnati Children's Hospital Medical Center, Cincinnati, Ohio, USA; 3 Department of Pediatrics, Washington University, St Louis, Missouri, USA

**Keywords:** nuclear receptor, phosphorylation, signal transduction, transcription regulation, mass spectrometry (MS), NR4A1, Nur77 mutants, IL-3, G-SCF, proximity labeling, DTT, dithiothreitol, FA, formic acid, HCD, high-energy collision-induced dissociation, MeCN, acetonitrile, MS1, mass spectra of peptide precursors, MS2, fragmentation mass spectrum of peptide from precursor ion, MWCO, molecular weight cutoff, nano-LC-MS, capillary liquid chromatography interfaced to a mass spectrometer, TCEP, Tris (2-carboxyethyl) phosphine, TFA, trifluoroacetic acid

## Abstract

The orphan nuclear receptor Nur77 is an immediate-early response gene that based on tissue and cell context is implicated in a plethora of cellular processes, including proliferation, differentiation, apoptosis, metabolism, and inflammation. Nur77 has a ligand-binding pocket that is obstructed by hydrophobic side groups. Naturally occurring, cell-endogenous ligands have not been identified, and Nur77 transcriptional activity is thought to be regulated through posttranslational modification and modulation of protein levels. To determine whether Nur77 is transcriptionally active in hematopoietic cells *in vivo*, we used an upstream activating sequence (UAS)-GFP transgenic reporter. We found that Nur77 is transcriptionally inactive *in vivo* in hematopoietic cells under basal conditions, but that activation occurs following cytokine exposure by G-CSF or IL-3. We also identified a series of serine residues required for cytokine-dependent transactivation of Nur77. Moreover, a kinase inhibitor library screen and proximity labeling-based mass spectrometry identified overlapping kinase pathways that physically interacted with Nur77 and whose inhibition abrogated cytokine-induced activation of Nur77. We determined that transcriptional activation of Nur77 by G-CSF or IL-3 requires functional JAK and mTor signaling since their inhibition leads to Nur77 transcriptional inactivation. Thus, intracellular cytokine signaling networks appear to regulate Nur77 transcriptional activity in mouse hematopoietic cells.

Nuclear receptor subfamily 4 group A member 1 (NR4A1; also termed Nur77/TR3/NGFIB) is an orphan member of the nuclear receptor superfamily. Nur77 has the typical structure of a nuclear receptor: an N-terminal transactivation domain, a conserved DNA-binding domain (DBD), and a C-terminal ligand-binding domain (LBD) with a C-terminal helix that provides ligand-dependent coactivator interactions (the AF2 domain) (1, 2). The transcriptional activity of most nuclear receptors depends on the presence *versus* absence of small-molecule ligands (3). However, the Nur77 ligand-binding pocket is filled by hydrophobic amino acid side chains, which should inhibit internal binding of a small molecule (2). Endogenous natural ligands that regulate Nur77 transcriptional activity have not been found, even if some studies have identified compounds (*i.e.*, cytosporone B, celastrol) that act as Nur77 agonists by binding to external surfaces on the LBD or antagonists (*i.e.*, diindolylmethane derivatives) by directly interacting with Nur77 LBD pocket (4, 5, 6). Because Nur77’s ligand-binding pocket is largely obstructed, alternative mechanisms may be relevant to provide natural, intracellular, temporal, and spatial control of Nur77 activity, and it is unknown in which tissues and under which conditions Nur77 might be transcriptionally active *versus* transcriptionally silent.

Nur77 is an immediate-early gene and an important transcription factor implicated in a plethora of cellular processes in response to different stimuli such as mitogens, cytokines, metabolic, and apoptotic signals (7, 8, 9, 10). Nur77 has been implicated in autoimmunity, regulation of T cell (Treg) differentiation, and T cell metabolism (11, 12, 13). Nur77 also plays central roles in the differentiation and survival of Ly6C− monocytes, and this subset of cells are absent in *Nur77*-deficient mice (14). In cancer cell lines and in stimulated thymocytes, phosphorylation of Nur77 induces exit from the nucleus and transfer to the mitochondria, where it promotes apoptosis by associating with Bcl-2 (15). Nur77 has been associated with mixed roles in oncogenesis. In blood-derived tumors (*i.e.*, leukemia and in lymphoma), Nur77 has been proposed as a tumor suppressor influencing key cellular processes such as inflammation and apoptosis (16, 17). In addition, murine deletion of the genes encoding for Nur77 and its homolog NR4A3 led to acute myeloid leukemia development (18). Conversely, in several solid tumors (*i.e.*, breast, colon, kidney, melanoma, and pancreas), it is overexpressed, and it acts as pro-oncogene promoting cell proliferation, survival, and migration/invasion while its inactivation by antagonists binding has shown to reduce tumor growth and survival (19, 20, 21, 22).

The molecular mechanisms that modulate Nur77 transcriptional activity remain poorly defined. In the present study, we aimed to define the *in vivo* distribution of active *versus* inactive Nur77. Surprisingly, we find that Nur77 is transcriptionally inactive in reporter assays *in vivo* in hematopoietic cells under basal conditions, but that transcriptional activation is induced by G-CSF. We show that cytokine-dependent transcriptional activation of Nur77 requires JAK and mTor signaling, and we identify a series of serine residues required for this activation in primary mouse hematopoietic cells, suggesting that intracellular signaling networks may modify Nur77 activity *via* posttranslational modification.

## Results

### Gal4-Nur77 reporter transcriptional activity is regulated by G-CSF *in vivo* and *in vitro*

We evaluated the expression of nuclear receptors in human AML bone marrow samples and noted that *NUR77* is one of the highest expressed nuclear receptors (Fig. S1*A*) (23). In addition, during human hematopoietic maturation, *NUR77* undergoes differential expression, with high expression in CD34+ stem/progenitor cells and low expression in promyelocytes, neutrophils, T-cells, and B-cells, but retained expression in monocytes (Fig. S1*B*). Similar patterns of expression could be observed in mouse hematopoietic cells, with expression in stem cells, reduced expression in progenitor cells, and the re-expression during neutrophil and monocyte maturation (Fig. S1, *C*–*E*) (24, 25). These patterns of differential expression across different hematopoietic cell types suggest that NUR77 may contribute to hematopoietic function and phenotypes in different hematopoietic cell types.

**Figure 1:**
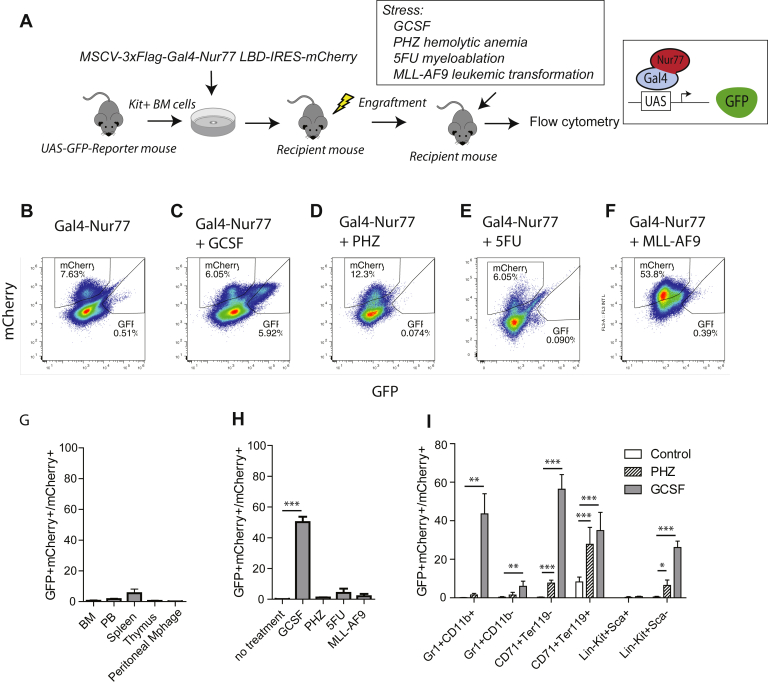
**Gal4-Nur77 transcriptional activity *in vivo*.***A*, schema for stem cell transplant procedure. *B*–*E*, Kit+ UAS-GFP bone marrow cells were transduced with MSCV-3xFlag-Gal4-Nur77 LBD-IRES-mCherry retroviruses and engrafted into sublethally irradiated recipient mice. Six weeks later, recipient mice were treated as indicated (G-CSF, PHZ, 5FU), and reporter activity was assessed (the ratio of mCherry+GFP+ *versus* total mCherry+ cells). *F*, Kit+ UAS-GFP bone marrow cells were transformed with MLL-AF9 retrovirus, transduced with Gal4-Nur77 retrovirus, and engrafted into recipient mice. *G*, summary ratios of GFP+mCherry+ cells relative to total mCherry+ cells in the bone marrow cells, peripheral blood, spleen, and peritoneal macrophages from mice transplanted with UAS-GFP bone marrow Kit+ cells transduced with Gal4-Nur77 (n = 5 recipient mice). *H*, ratio of GFP+mCherry+ cells relative to total mCherry+ cells in bone marrow cells from mice transplanted with UAS-GFP bone marrow Kit+ cells transduced with Gal4-Nur77 and treated as indicated (n = 5 recipient mice per group). *I*, reporter activity was evaluated by flow cytometry in hematopoietic subpopulations as indicated. ∗*p* < 0.05. ∗∗*p* < 0.01. ∗∗∗*p* < 0.001, *t* test with Welch’s correction.

To determine whether Nur77 activity might be transcriptionally active *versus* inactive in different cell types *in vivo*, we used a previously characterized *in vivo* nuclear receptor reporter assay using UAS-GFP transgenic mice (3, 26). Briefly, UAS promoter sequences are recognized by the yeast Gal4 transcription factor and are not activated by mammalian proteins. We generated a retrovirus expressing the recombinant protein Gal4-Nur77 where the modular *Gal4* DBD (Gal4-DBD) is fused to the *Nur77* LBD (Nur77-LBD) (MSCV-Flag-Gal4 DBD-Nur77 LBD-IRES-mCherry). UAS-GFP bone marrow Kit+ cells were transduced with Gal4-Nur77 and then transplanted into lethally irradiated recipient mice (Schema Fig. 1*A*). Using this system, the reporter specifically responds when intracellular ligands or other stimuli are present that enable transactivation by Nur77-LBD. Six weeks after transplantation, the recipient mice were sacrificed and bone marrow, peripheral blood, spleen, thymus, and peritoneal macrophages were collected and analyzed by flow cytometry. We did not observe GFP^+^mCherry^+^ cells in any of these five tissue types, suggesting that under basal conditions, Nur77 may be transcriptionally inactive (or potentially repressive) in hematopoietic cells *in vivo* (Fig. 1*B* with summary data in Fig. 1*G*). We evaluated the effect of hematopoietic stress on Nur77 transcriptional activity *in vivo*. We treated transplanted mice (UAS-GFP x Gal4-Nur77) with granulocyte-colony stimulating factor (G-CSF) to induce granulopoiesis, phenylhydrazine (PHZ) to induce hemolytic anemia, and subsequent erythropoiesis or 5-fluorouracil (5FU) to induce myeloablation and subsequent stem/progenitor expansion. We further evaluated a myeloid malignant stressor, the engraftment of MLL-AF9 leukemia cells (27). Following these stressors, the mice were sacrificed and reporter activity compared with the mice engrafted under basal conditions. We observed an increase in the proportion of GFP+mCherry+ cells in total bone marrow cells following G-CSF treatment, but not following treatment with other stressors (Fig. 1, *C*–*F* and *H*). Gal4-Nur77 reporter activity was not uniform across all cell types following G-CSF treatment, and GFP+mCherry+ cells were enriched in Gr1+CD11b+, CD71+Ter119+, and CD71+Ter119-cells (Fig. 1*I*). Thus, Gal4-Nur77 appears to be broadly transcriptionally inactive in normal and malignant hematopoietic cells under basal conditions, but it becomes transcriptionally active during G-CSF-induced granulopoiesis.

### G-CSF and IL-3 regulate Gal4-Nur77 transcriptional activity *via* JAK1/JAK2 pathways *in vitro*

**Figure 2:**
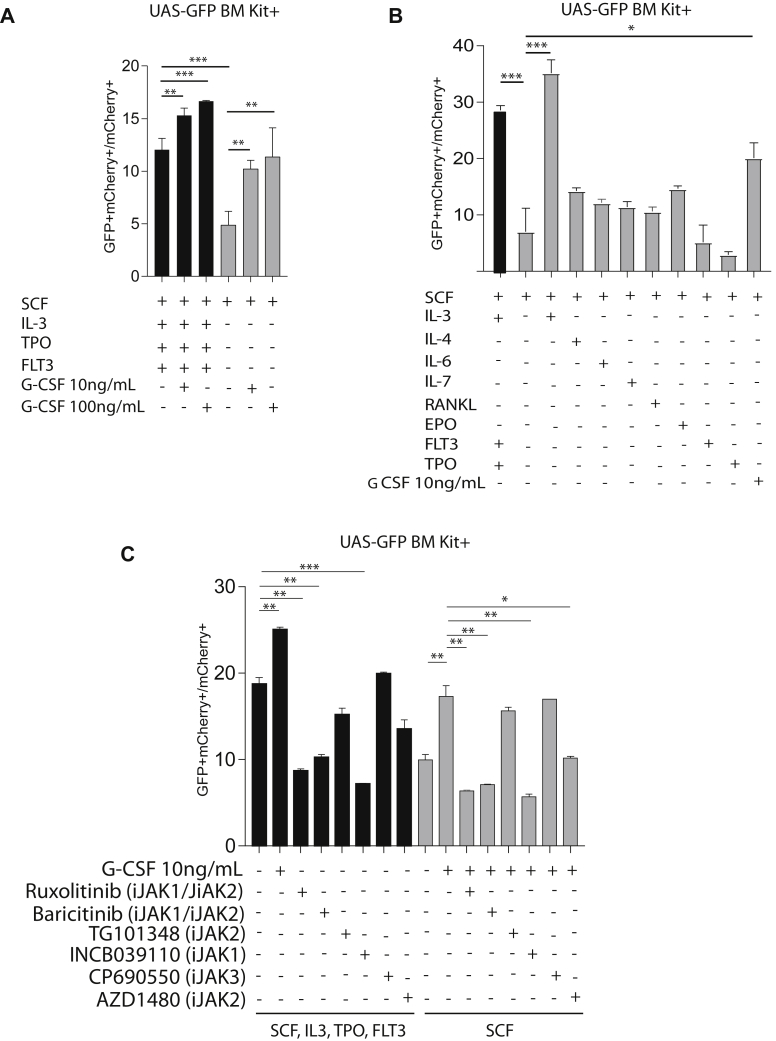
**JAK1/JAK2 pathway regulates Gal4-Nur77 transcriptional activation *in vitro*.***A*–*C*, ratio of GFP+mCherry+ cells relative to total mCherry+ cells in UAS-GFP bone marrow Kit+ cells transduced with Gal4-Nur77 and treated as indicated for 24 h. *C*, the JAK1/JAK2 inhibitors were used at the following concentrations:Ruxolitinib 0.2 μM, Baricitinib 0.2 μM, TG101348 0.3 μM, INCB039110 0.2 μM, CP690550 20 nM and AZD1480 1 μM. Each experimental point was performed in triplicate. ∗∗*p* < 0.05. ∗∗*p* < 0.01. ∗∗∗*p* < 0.001, *t* test with Welch’s correction.

We assessed the effect of G-CSF on Gal4-Nur77 reporter activity *in vitro* using UAS-GFP BM Kit+ cells transduced with Gal4-Nur77. UAS-GFP BM Kit+ cells were grown in two different cytokine cocktails: a minimal media containing only stem cell factor (SCF) and in a supplemented transplant media (TM) containing SCF, interleukin-3 (IL-3), thrombopoietin (TPO), and fms-related tyrosine kinase 3 ligand (FLT3). We observed that Gal4-Nur77 reporter activity was augmented by G-CSF under both conditions and that basal reporter activity was greater in the cytokines-enriched TM compared with minimal media (Fig. 2*A*). To determine whether a particular cytokine was responsible for reporter transactivation by Gal4-Nur77, UAS-GFP BM Kit+ cells were transduced with Gal4-Nur77, cultured in minimal media, and exposed to individual cytokines (IL-3, IL-4, IL-6, IL-7, receptor activator of nuclear factor kappa-B ligand-RANKL, erythropoietin-EPO, FLT3L and TPO). Complete TM and G-CSF were used as positive controls. In addition to G-CSF, we found that IL-3 augmented Gal4-Nur77 reporter activity (Fig. 2*B*).

Both G-CSF and IL-3 are activators of the Janus Kinase (JAK)-Signal Transducers and Activator of Transcription (STAT) signaling pathway and regulate hematopoietic progenitor cell proliferation and differentiation (28). The JAK family is composed of four members (JAK1, 2, 3, and TYK2) with differential association with specific cytokine receptors (29). To assess whether G-CSF and IL-3 regulation of Gal4-Nur77 reporter activity involve overlapping JAK signaling, JAK inhibitors were administrated to UAS-GFP BM Kit+ cells. Ruxolitinib (iJAK1/2), baricitinib (iJAK1/2), and INCB039110 (iJAK1) inhibited IL-3 and G-CSF mediated reporter transactivation by Gal4-Nur77 (Fig. 2*C*), whereas CP690550 (iJAK3) was not effective. Thus, in hematopoietic cells, Gal4-Nur77 transcriptional activity is regulated *via* IL-3, in addition to G-CSF, and depends on signaling through JAK1 and/or JAK2.

### Gal4-Nur77 mutational analysis and effects on transcriptional activity

**Figure 3:**
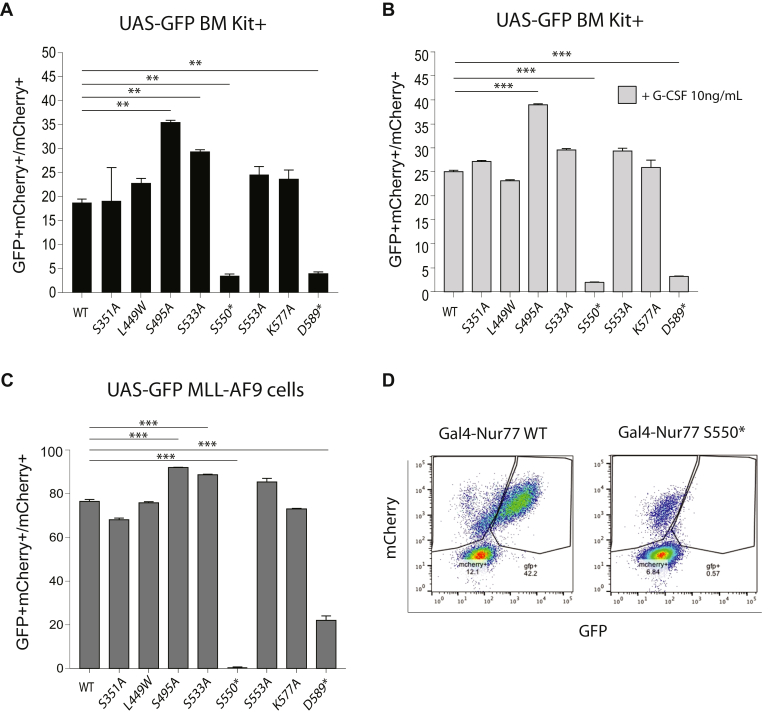
**Analysis of transcriptional activity of Nur77 mutants *in vitro*.***A* and *B*, UAS-GFP bone marrow Kit+ transduced with Gal4-Nur77 or indicated mutations were treated without and with G-CSF and mCherry+GFP+ cells quantified. *C*, UAS-GFP MLL-AF9 cells transduced with Gal4-Nur77 or indicated mutations and treated as indicated for 24 h. *D*, representative data showing GFP and mCherry intensity in UAS-GFP MLL-AF9 cells transduced with Gal4-Nur77 or Gal4-Nur77 S550∗. Each experimental point was performed in triplicate. ∗∗*p* < 0.01. ∗∗∗*p* < 0.001, *t* test with Welch’s correction.

We evaluated whether specific posttranslational modification sites were necessary for Gal4-Nur77 transcriptional activity. We generated a series of mutations in the Nur77 LBD at sites previously implicated in Nur77 posttranslational modification. These included: S351, which can be phosphorylated by Akt and is crucial for Nur77 tumor suppression activity (30, 31); L449W, which affects optimal occupation of an acyl chain in the bulky binding pocket and is required for the optimal trihydroxybenzene potency (32); S533, which can be phosphorylated by Akt2 (32); S553A, which alters the native surface feature of the LBD (33); and K577A, which alters a canonical SUMOylation consensus motif within the LBD (34). In addition, we generated the phosphorylation mutant S495A based on its strategic position within the structure of Nur77 LBD, and two deletion mutants of the ligand-dependent activation function 2 (AF-2) domain (S550∗ and D589∗). The protein expression of the mutants was verified on Western blot (Fig. S2*A*). UAS-GFP BM Kit+ cells were again transduced with Gal4-Nur77, and transcriptional activation was evaluated after culture in TM for 24 h with and without G-CSF treatment by the percentage of mCherry+GFP+ cells (Fig. 3, *A* and *B*). In addition, UAS-GFP bone marrow cells transformed with MLL-AF9 (which maintain a monocytic morphology) retrovirus can be cultured *ex vivo* in media containing IL-3. These cells are highly dependent on IL-3 for growth and survival and exhibited consistent reporter transactivation by Gal4-Nur77 under *ex vivo* conditions (Fig. 3, *C* and *D*). In both cellular contexts, S495A augmented Gal4-Nur77 reporter activity, and both AF2 deletions abrogated Nur77 reporter activity. S533A was hyperactive, and S351A, L449W, S553A, and K577A did not result in significant differences compared with wild-type Gal4-Nur77 in either cell context.

We assessed the effect of JAK inhibitors on the activity of Gal4-Nur77 LBD WT, S351A, S495A, K577A, and D589∗ mutations in MLL-AF9 leukemia cells. Ruxolitinib and baricitinib were again effective at reducing the reporter activity, with greater effect on the median fluorescence intensity (MFI) of GFP than the proportion of GFP+ cells, as might be expected in a system with high basal GFP expression (Fig. S2, *B*–*F*). This inhibition was observed with S351A, S495A, and K577A mutations (Fig. S2, *C*–*E*), and activity was again absent with D589∗ (Fig. S2*F*). Thus, the AF2 domain appears necessary for Gal4-Nur77 transactivation, and S495 and S533 both augment Gal4-Nur77 activity, but it retains sensitivity to JAK inhibitors. In addition, we performed a nuclear/cytoplasmic fractionation in MLL-AF9 cells expressing Gal4-Nur77 to determine if JAK inhibitors sensitivity could be caused by a shift in Gal4-Nur77 reporter cellular localization. We observed that Gal4-Nur77 47 kDa band was present in both the nucleus and cytosol, while the presence of additional Gal4-Nur77 33 kDa and 20 kDa truncated bands were nuclear exclusive. JAK inhibitors administration did not alter the baseline patterns of cytosolic and nuclear localization (Fig. S2*G*).

We examined variant distribution within the human population as represented in the gnomAD database (gnomad.broadinstitute.org). Within Nur77 (P22736), there are 69 serines, 18 of which are found in the LBD. However, of the 346 reported variants involving serine residues, only 12 occur within the LBD (*p* < 0.001) (S380P: 1; S454L: 3; S498G: 6; S546T: 2). Likewise, of 27 total lysine residues, 11 are found in the LBD but only 32 of 414 variant lysine alleles are found in LBD lysines (*p* < 0.001). This suggests that additional LBD serine and lysine positions may be biologically relevant and preferentially conserved.

**Figure 4:**
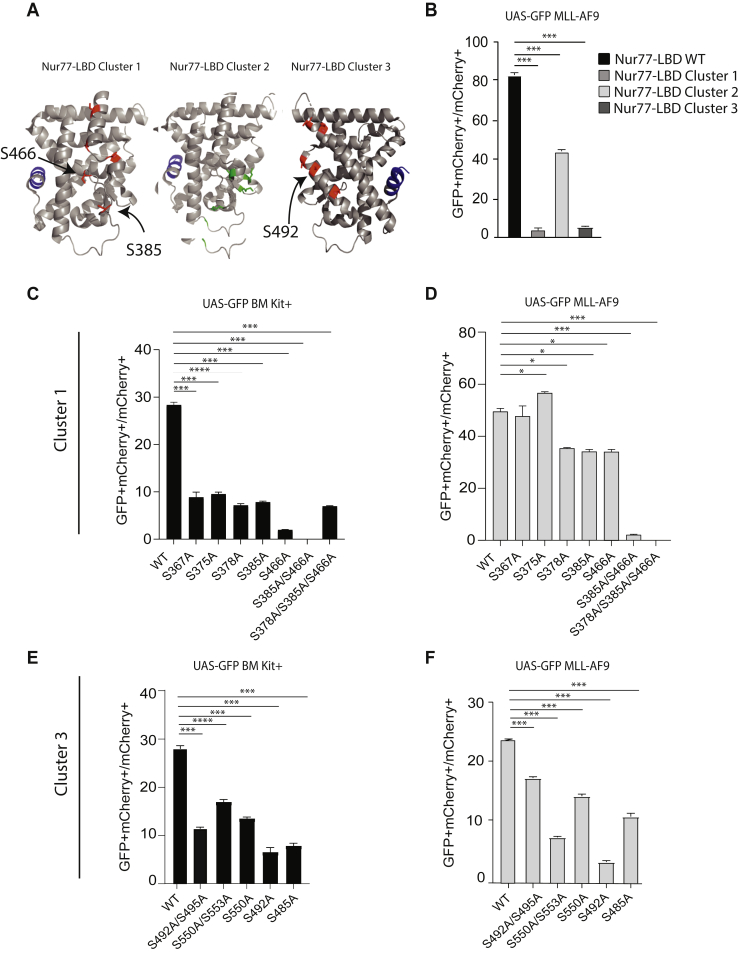
**Analysis of transcriptional activity of Gal4-Nur77 phosphorylation cluster mutants, *in vitro*.***A*, structure of the Nur77 ligand-binding domain (PDB 3V3E). Serines mutated in clusters 1 and 3 are highlighted in *red*, lysine mutated in cluster 2 is *green*, and the AF2 domain from D589 is highlighted in *blue*. *B*, ratio of GFP+mCherry+ cells relative to total mCherry+ cells in UAS-GFP MLL-AF9 cells transduced with Gal4-Nur77 or cluster mutations. *C* and *E*, ratio of GFP+mCherry+ cells relative to total mCherry+ cells in UAS-GFP bone marrow Kit+ and *D* and *F*, UAS-GFP MLL-AF9 cells transduced with Gal4-Nur77, or the indicated mutants. Each experimental point was performed in triplicate ∗*p* < 0.05. ∗∗*p* < 0.01. ∗∗∗*p* < 0.001, *t* test with Welch’s correction.

We examined the structure of the Nur77 LBD (PDB 2QW4) and defined three clusters of external-facing possible posttranslational modification sites: cluster 1 (S367, S375, S378, S385, and S466), cluster 2 (K381, K386, K397, K456, and K361), and cluster 3 (S485, S492, S495, S550, and S553) (Fig. 4*A*, clusters 1 and 3 represent serine residues along two different shared surfaces, and cluster 2 was a combination of externally facing lysines). Expected expression and retained nuclear/cytoplasmic localization patterns of each cluster mutant were verified on Western blot (Fig. S3, *A* and *B*). The cluster mutants were transduced in UAS-GFP MLL-AF9 cells and the transcriptional activation was evaluated at 24 h by the percentage of mCherry+GFP+ cells. Cluster 1 and 3 mutants abrogated transactivation by the Gal4-Nur77 reporter in both MLL-AF9 cells and in Kit+ bone marrow cells, whereas cluster 2 retained some activity (Figs. 4*B* and S3*C*). To define which specific residues within clusters 1 and 3 were responsible for Gal4-Nur77 transcriptional activity, we generated a series of single and combination mutants, and protein expression of these mutants was verified on Western blot (Fig. S3, *D* and *E*). These mutants were transduced in UAS-GFP BM Kit+ and UAS-GFP MLL-AF9 cells, and the transcriptional activation was evaluated at 24 h by the percentage of mCherry+GFP+ cells. Cluster 1 was fractionated into seven different vectors (S367A, S375A, S378A, S385A, S466A, S385A/S466A, and S378A/S385A/S466A). Although S367A and S375A inhibited transactivation in Kit+ cells, they had little effect in MLL-AF9 cells. In both contexts, S378A, S385A, S466A, and S378A/S385A/S466A decreased reporter transactivation, and S385A/S466A abrogated activity, suggesting a role for these two phosphorylation sites within the Nur77-LBD (Fig. 4, *C* and *D*). Cluster 3 was deconvoluted using five different vectors (S492A/S495A, S550A/S553A, S550A, S492A, and S485A). All the mutants from cluster 3 displayed reduced reporter transactivation in both cell types, and no single mutation completely abrogated Gal4-Nur77 reporter activity (Fig. 4, *E* and *F*).

### Kinase inhibitor screening and Gal4-Nur77 transcriptional activity

**Figure 5:**
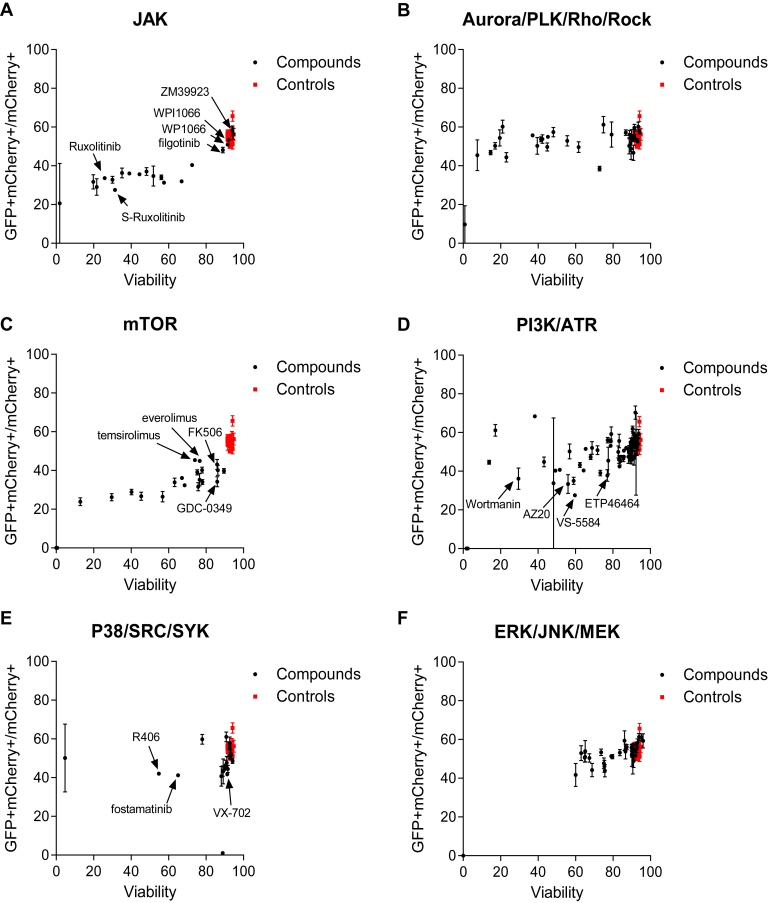
**Kinase inhibitors library screening.** UAS-GFP MLL-AF9 cells were transduced with Gal4-Nur77 and immediately treated with 1 μM concentrations of each compound for 24 h in duplicate. Cell viability determined by Hoechst 33342 staining. Compounds were organized by pathways: (*A*) JAK, (*B*) Aurora/PLK/Rho/Rock, (*C*) mTOR, (*D*) P38/SRC/SYK, (*E*) PI3K/ATR, (*F*) ERK/JNK/MEK.

To determine whether and which specific kinases might be involved in cytokine signal transduction that regulate Gal4-Nur77 activity, we performed a screening assay using a library of 436 kinase inhibitors. Compounds were organized by principle pathways. We again noted effects by broad JAK inhibitors, with limited effects observed among compounds with weak JAK2 activity and no effects in JAK3 specific inhibitors (Fig. 5*A*). The MLL-AF9 leukemia cells are highly dependent on IL-3, and it is not surprising that viability declined to some extent among compounds in this class. However, in this assay, reduced cell viability was not synonymous with reduced reporter activity, as diverse inhibitors of Aurora, polo-like, Rho, and ROCK kinases lead to reduced cell viability without reduced GFP output (Fig. 5*B*). Multiple inhibitors of the mTor pathway inhibited reporter activity (Fig. 5*C*). PI3K and ATR inhibitors also were active, although generally, these were compounds with cross-inhibitory effects on mTor and not compounds with ATR specificity (Fig. 5*D*). The multikinase inhibitor R406 and its prodrug fostamatinib were both active, whereas ERK, JNK, and MEK inhibition showed minimal effects (Fig. 5, *E* and *F*). Inhibition of other pathways did not inhibit Gal4-Nur77 reporter activity (ABL, ALK, ATM, BTK, CDK, EGFR/HER2, FAK, IGFR/PDGFR, Kit/Mek, NFkB, TGFb, VEGFR, Figs. S4, *A*–*F* and S5, *A*–*F*). Thus, multiple kinases and pathways of kinases appear to intersect with cytokine-induced signal transactivation of Gal4-Nur77.

### TurboID-based proximity labeling

**Figure 6:**
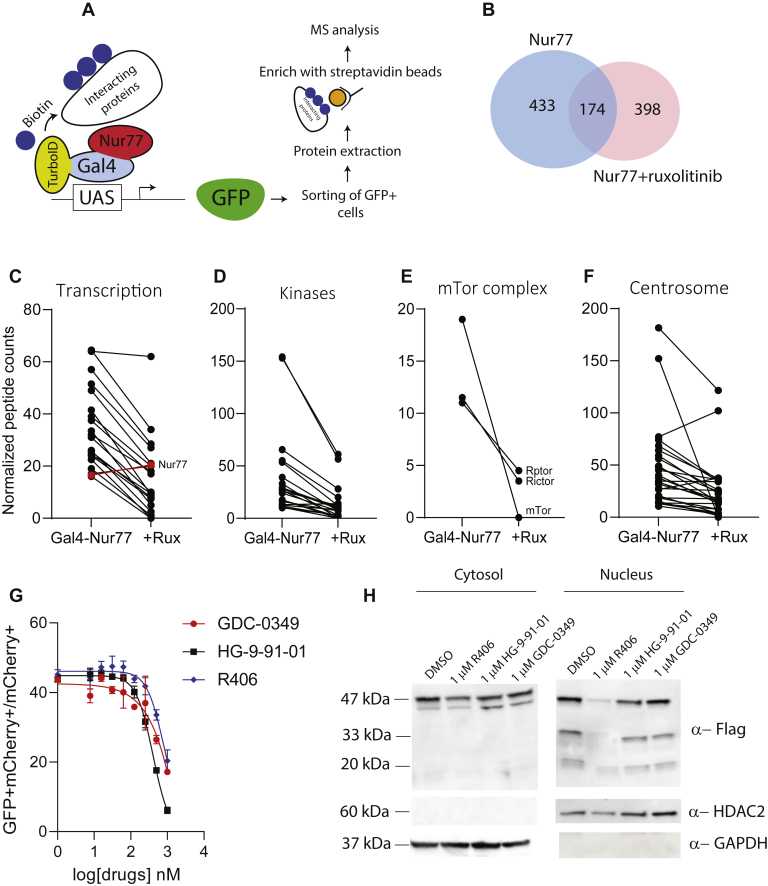
**Proximity labeling of Gal4-Nur77 in the presence *versus* absence of Ruxolitinib.***A*, schema of TurboID mechanism in UAS-GFP MLL-AF9 cells. *B*, Venn diagram showing the interactors of Gal4-Nur77 identified in presence *versus* absence of ruxolitinib; all interaction proteins had a quantitative Value (Normalized Total Spectra) greater than 10 after the control background subtraction and a *p* value <0.01. Results were organized by protein molecular function using the GO enrichment analysis tool: (*C*) Transcription, (*D*) Kinases, (*E*) Centrosome, (*F*) mTOR. *G*, ratio of GFP+mCherry+ cells relative to total mCherry+ cells in UAS-GFP MLL-AF9 cells transduced with Gal4-Nur77 and treated as indicated for 24 h. *H*, nuclear and cytosolic protein lysate of UAS-GFP MLL-AF9 cells transduced with MSCV-3xFlag-Gal4-Nur77 LBD-IRES-mCherry, treated as indicated for 24 h and analyzed by Western blot using anti-Flag antibody. HADC2 and GAPDH were used as a loading control for nuclear and cytosolic extracts, respectively.

To identify proteins that bind to and might modify Nur77 to induce cytokine-dependent Nur77 activity, we used proximity labeling. A promiscuous biotin ligase (TurboID) was fused to Gal4-Nur77 to enable biotinylation of proximal proteins (∼10 nm, schema Fig. 6*A*) (35). The TurboID-Gal4-Nur77 fusion was transduced into UGN x MLL-AF9 leukemia cells and GFP+ cells were sorted and expanded. These cells were then treated with ruxolitinib *versus* control and excess biotin was added. After 16 h of incubation, biotinylated proteins were captured by streptavidin-based affinity purification followed by mass spectrometry (MS) analysis (Fig. 6*A*). An aliquot of biotinylated proteins was analyzed by immunoblotting to confirm their usability for subsequent MS analysis (Fig. S6*A*). Cells expressing TurboID-IRES-mCherry were compared to identify nonspecific biotinylation, and the peptide counts from these samples were subtracted as background from the TurboID-Gal4-Nur77 samples. We examined the reproducibility of the data and found high Pearson correlation values among the biological replicates of both datasets (Nur77 r > 0.98 and Nur77+ruxolitinib r > 0.94). We designated proteins as enriched interactors of Gal4-Nur77 if they had a quantitative value (Normalized Total Spectra) greater than 10 after the control background subtraction and a *p* value <0.01. These analyses identified 433 Gal4-Nur77-enriched interactors, 398 Gal4-Nur77-enriched interactors in presence of ruxolitinib, and 174 that were present in both conditions (Fig. 6*B*). Results were organized by protein molecular function using the GO enrichment analysis tool (36). Broad sets of transcription factors were enriched in the Gal4-Nur77 samples. Of these, all but Nur77 and Cnot1 were reduced with ruxolitinib treatment (Fig. 6*C*). We identified a series of kinases that were enriched in Gal4-Nur77. Nearly uniformly, these were depleted in ruxolitinib-treated samples, with the exception of the kinases Phkb and Pgk1, which were enriched in the presence of ruxolitinib (Figs. 6*D* and S6*B*). Only five phosphatases were identified, three of which (Ppp6r3, Ankrd28, Ankrd52) were enriched in the Gal4-Nur77 group and depleted in ruxolitinib, whereas Inpp5d and Ankrd44 were more abundant in the presence of ruxolitinib (Fig. S6*C*). Three members of mTOR pathway were specifically enriched in Nurr77 and depleted in the presence of ruxolitinib: mTOR (Serine/threonine-protein kinase mTOR), Rictor (Rapamycin-insensitive companion of mTOR), and Rptor (Regulatory-associated protein of mTOR) (Fig. 6*E*). Unexpectedly, diverse members of the centrosome complex, myosin complex, and ubiquitin complex were identified and again were broadly reduced in the ruxolitinib-treated samples (Figs. 6*F* and S5, *D* and *E*). Conversely, members of 14-3-3, chaperone and ribosome family were enriched in the presence of ruxolitinib (Fig. S6, *F*–*H*).

Kinases identified in proximity labeling were examined for overlap with results from the kinase inhibitor screen (Fig. 5). Overlap was identified in mTor pathway and in the SYK/polykinase inhibitor R406 (potential R406 targets identified in the proximity labeling include Mylk, Ikbke, Pak2, Lrrk2, Slk, Ripk1, Sik2, and Gak). Compounds with target specificity were sought, and three compounds were selected for further validation: GDC-0349 (mTor inhibitor), HG-9-91-01 (SIK1/SIK2 inhibitor), and R406 (SYK/polykinase inhibitor). These three compounds each inhibited the transcriptional activity of the Gal4-Nur77 reporter, albeit at higher concentrations than might be required for simple enzyme inhibition (Fig. 6*G*). We further evaluated cytosolic *versus* nuclear localization of Gal4-Nur77 during treatment with these compounds. Interestingly, we observed a reduction of Gal4-Nur77 protein expression in the nuclear compartment upon R406 administration, suggesting that Gal4-Nur77 sensitivity to this SYK inhibitor may be related to shifts in the intracellular localization of Gal4-Nur77 (Fig. 6*H*).

## Discussion

Nur77 has been proposed as a true orphan nuclear receptor. Unlike most other nuclear receptors, Nur77 does not appear to bind to natural ligands. Rather, control of its transcriptional effects has been proposed to occur through regulation of expression, protein degradation, and subcellular localization (37). In addition, multiple posttranslational modifications have been described in diverse cell types, including phosphorylation, acetylation, and SUMOylation (37, 38, 39, 40). Sites of posttranslational modification have been identified in both the N-terminal transactivation domain, the DBD, and the LBD. Moreover, multiple enzymes have been identified that can posttranslationally modify Nur77, including AKT1 (with specific activity at S351), MAPK, CK2alpha, and Pin1 (41, 42, 43, 44).

In our study, we applied retroviral expression of a widely used UAS/Gal4 nuclear receptor transactivation reporter assay. This assay reads out isolated Nur77-LBD transactivation activity in the context of a chimeric fusion with the Gal4-DBD. This has the advantage of isolating and specifically interrogating the function and regulation of the Nur77-LBD in primary murine hematopoietic cells, but it does not integrate the intracellular regulation of the Nur77-DBD or the local promoter/enhancer chromatin contexts of Nur77 target genes. Unexpectedly, we found that under basal conditions, the Gal4-Nur77 was transcriptionally inactive across broad tissue types *in vivo*, but that stimulation with myeloid cytokines led to Gal4-Nur77 transcriptional activation. The overlapping JAK-dependent effects of G-CSF and IL-3 suggested posttranslational effects involving signal transduction kinases. We tested a series of previously defined phosphorylation sites but found mutations at these sites had little effect on transcriptional activation of Gal4-Nur77 (S351A, L339W, S553A, K577A). In particular, AKT1 and MAPK had been implicated as kinases at S351 (39, 45). However, a mutation at this site did not alter transactivation, and we did not observe consistent effects of AKT or MAPK/ERK/MEK inhibitors in a kinase screen (Figs. 3 and 5). Using a screening approach, we identified a series of serines that appear necessary for Gal4-Nur77 activation by G-CSF and IL-3 that have not been previously implicated in the regulation of Nur77.

We previously noted activation of the UAS-GFP reporter by G-CSF *in vivo* in bone marrow cells transduced with Gal4-RXRA (3), raising the possibility of nonspecific activation of the UAS-GFP reporter by cytokine signal transduction. During initial characterization of the UAS-GFP reporter, we observed that AF2 deletions in Gal4-RARA and Gal4-RARG abrogated *in vitro* and *in vivo* responses to all-trans retinoic acid (26) and that deletion of the Gal4-RXRA AF2 domain abrogated *in vivo* response to both bexarotene and G-CSF signaling, suggesting reporter specificity and dependence of the AF2 domain among retinoid receptors. Likewise, in the case of Gal4-Nur77, we again observed signal reduction with AF2 deletion (Fig. 3*C*), in addition to effects seen with a series of point mutations (Figs. 3 and 4). Several differences also are noted in cytokine-dependent effects on UAS/Gal4 reporters. *In vitro*, Gal4-RARA and Gal4-RXRA are inactive in MLL-AF9 cells grown in IL-3 containing media (27), whereas Gal4-Nur77 is consistently active (Fig. 3). *In vivo*, both G-CSF and PHZ stressors induced Gal4-RXRA reporter activity, whereas activity of Gal4-RARA was absent, and Gal4-Nur77 was not induced following PHZ (Fig. 1, *D* and *H*) (46). Leukocytosis is noted during recovery from PHZ hemolytic anemia, and this may provide a potential mechanism of shared effects between G-CSF and PHZ stressors. The mechanism of Gal4-RXRA activation by G-CSF and PHZ appears to be *via* noncell intrinsic increases in serum natural ligands (C24:5 and DHA), whereas G-CSF-induced activation of Gal4-Nur77 appears to be cell-intrinsic and involves intracellular JAK and mTor signaling. Thus, multiple nonresponsive mutations have been inactive in the UAS-GFP reporter, suggesting specificity, and RXRA and Nur77 appear to respond to overlapping cytokine signals *via* unrelated mechanisms.

Not all studies have found that Nur77 transactivation depends on the AF2 domain. For example, Nur77 transcriptional activation by the mutant Ca^2+^/CaM-dependent protein kinase kinase (CaMKIV) was AF2 independent, and replacement with the thyroid receptor AF2 domain retained similar activity (47). In contrast, G-CSF/IL-3-dependent cellular signaling was abrogated by deletions of AF2 (Fig. 3), suggesting necessary interactions with more canonical coactivator machinery. However, proximity labeling studies identified interactions with proteins involved in mRNA transcription (Eif4g2, Eif4enif, Eif4b, Gigyf2, Ankhd1, Elp3, Ttf2) and several helicases (Ascc3, Dna2, Helz), but no clear interactions with canonical coactivator/corepressors that typically interact with nuclear receptors *via* the AF2 domain (*e.g.*, SWI/SNF components, p300, CBP, Ncor1, or Ncor2).

Although several kinases have been previously implicated in Nur77 posttranslational modifications (*e.g.*, Akt1, Mapk, Pin1, Ck2alpha), we did not observe interactions with members of these kinase families in a kinase inhibitor screen or during proximity labeling. Kinase inhibition and proximity labeling displayed overlapping phenotypes. Of note, we could validate effects of JAK, mTor, and Sik using inhibitors with high specificity (baricitinib, INCB039110, HG-9-91-01, and GDC-0349), suggesting that multiple signal transduction pathways may converge to regulate Nur77 transactivation in response to cellular stimuli. Nur77 has not been implicated in centrosome function. Its localization is canonically nuclear, and translocation to the cytoplasm and mitochondria is associated with apoptosis (48). During proximity labeling, we identified interactions with multiple centrosome proteins (Nin, Dock2, Cep350, Cep192, Cep170, Cep131, Cep152, Cep97, Cep135, Cep72). In a database of proximity labeling studies (https://reprint-apms.org/) these proteins are rarely detected; all but Cep170 being reported in fewer than 7% of studies. Therefore, it is unclear whether this represents an unexpected effect of the TurboID-Gal4-Nur77 construct or a cellular function of Nur77. These interactions are unlikely to modulate the transactivation potential of Nur77 and therefore, were not pursued further as part of this study. Our proximity labeling study also identified members of 14-3-3 family as Nur77 interactors, and a recent publication confirmed that Nur77 binds 14-3-3 to facilitate 14-3-3-dependent YAP ubiquitination/degradation and to activate the Hippo signaling pathway (31). In summary, using a nuclear receptor reporter assay, we find that the LBD of Nur77 is transcriptionally inactive across diverse hematopoietic cell types under basal conditions *in vivo*, but that cytokines with overlapping signal transduction pathways (G-CSF and IL-3) lead to transcriptional activation of Nur77 in multiple myeloid cell types. We implicate JAK, mTor, and Sik signaling in these pathways, find that multiple Nur77 serine sites may be sites for posttranslational regulation, and that this signaling requires the AF2 domain. This adds additional kinases and serine sites to the already long list of sites modified on Nur77 and further suggests that Nur77 activity may be regulated by posttranslational modification and not simply by control of mRNA and protein levels.

## Experimental procedures

### Reagents and constructs

Anti-FLAG antibody (M2) was from Sigma-Aldrich, HDAC2 (2540S) was from Cell Signaling, and GAPDH Antibody (FL-335) was from Santa Cruz Biotechnology. PHZ and 5-FU were from Sigma-Aldrich. G-CSF was from Amgen. Cytokines were purchased from R&D Systems. The following antibodies were used for flow cytometry: CD11b (BD Biosciences, Clone M1/70), Gr1 (eBioscience, Clone RB6-8C5), and (BD Biosciences, Clone AL-21). Fluorescence was detected on a ZE5 Cell Analyzer (Biorad) CD71 (eBioscience, Clone R17217), Sca-1 (eBioscience, clone D7) Ter119 (eBioscience, TER119), c-Kit (eBioscience, clone 2B8). Biotin was from Sigma-Aldrich (B4501). Ruxolitinib, baricitinib, TG101348, INCB039110, CP690550 and AZD1480 were a gift from Jaebok Choi’s laboratory. All the constructs MSCV-3xFlag-Gal4-Nur77 LBD-Ires-mCherry, MSCV-3xFlag-Gal4-Nur77 LBD(S351A)-Ires-mCherry, MSCV-3xFlag-Gal4-Nur77 LBD(L449W)-Ires-mCherry, MSCV-3xFlag-Gal4-Nur77 LBD(S495A)-Ires-mCherry, MSCV-3xFlag-Gal4-Nur77 LBD(S533A)-Ires-mCherry, MSCV-3xFlag-Gal4-Nur77 LBD(S550∗)-Ires-mCherry, MSCV-3xFlag-Gal4-Nur77 LBD(S553A)-Ires-mCherry, MSCV-3xFlag-Gal4-Nur77 LBD(K577A)-Ires-mCherry, MSCV-3xFlag-Gal4-Nur77 LBD(D589∗)-Ires-mCherry, MSCV-3xFlag-Gal4-Nur77 LBD (S367A/S375A/S378A/S385A/S466A) -Ires-mCherry, MSCV-3xFlag-Gal4-Nur77 LBD (K381A/K386A/K397A/K456A/K461A)-Ires-mCherry, MSCV-3xFlag-Gal4-Nur77 LBD(S485A/S492A/S495A/S550A/S533A)-Ires-mCherry, MSCV-3xFlag-Gal4-Nur77 LBD(S367A)-Ires-mCherry, MSCV-3xFlag-Gal4-Nur77 LBD(S375A)-Ires-mCherry, MSCV-3xFlag-Gal4-Nur77 LBD(S378A)-Ires-mCherry, MSCV-3xFlag-Gal4-Nur77 LBD(S385A)-Ires-mCherry, MSCV-3xFlag-Gal4-Nur77 LBD(S466A)-Ires-mCherry, MSCV-3xFlag-Gal4-Nur77 LBD(S385A/S466A)-Ires-mCherry, MSCV-3xFlag-Gal4-Nur77 LBD(S378A/S385A/S466A)-Ires-mCherry, MSCV-3xFlag-Gal4-Nur77 LBD(S492A/S495A)-Ires-mCherry, MSCV-3xFlag-Gal4-Nur77 LBD(S550A/S553A)-Ires-mCherry, MSCV-3xFlag-Gal4-Nur77 LBD(S550A)-Ires-mCherry, MSCV-3xFlag-Gal4-Nur77 LBD(S492A)-Ires-mCherry, MSCV-3xFlag-Gal4-Nur77 LBD(S485A)-Ires-mCherry, MSCV-TurboID and MSCV-TurboID-3xFlag-Gal4-Nur77 LBD were generated in our laboratory.

### Retrovirus production and transduction

7 × 10^6^, 293T/17 cells were seeded in a 150 cm^2^ dish in DMEM (high glucose) + 10% FBS +1% Glutamax, 18 to 24 h before transfection and grown to 80% confluence. In total, 90 μl DNA 1 μg/μl, 64.5 μl DNA EcoPac (Retro pkg), 1 μg/μl, and 3.75 ml DMEM were mixed. In total, 120 μl LF3K or LF2K (Lipfectamine) and 3.75 ml DMEM were mixed. The two mixtures were incubated for 5 min, then mixed together and incubated for 15 min. In total, 2.5 ml of the mixture was dropped-wise onto the 293T/17 cells. Fresh medium was changed after 18 to 24 h transfection. Virus was collected at 48 h and 72 h and concentrated with Lenti-X Concentrate (PEG-Salt soln.) to filtered virus. The Virus was resuspended in DMEM and stored at −80 °C.

### Mice

Mice were maintained in a specific pathogen-free barrier facility maintained on a 12 h light–dark cycle. Upon weaning, all mice were group housed, up to five mice of the same sex per cage. Food and water bottle were provided in a recess of the metal wire lid at the top of the cage. Cages were changed once every week. Six to ten week (C57Bl/6 background) old mice were typically used for experimentation. Equal numbers of male and female mice were used, no gender biases were noted.

### Hematopoietic cell culture

UAS-GFP mice bone marrow Kit+ cells were isolated using an Automacs Pro (Miltenyl Biotec) per manufacture’s protocol. Kit+ cells were plated in progenitor expansion medium (RPMI1640 medium, 15% FBS, Scf (50 ng/ml), IL3 (10 ng/ml), Flt3L (25 ng/ml), Tpo (10 ng/ml), L-glutamine (2 mM), sodium pyruvate (1 mM), HEPES buffer (10 mM), penicillin/streptomycin (100 units/ml), β-mercaptoethanol (50 μM)). UAS-GFP x MLL-AF9 cells were produced as described and cultured *in vitro* using similar media, but without Flt3L, or Tpo.

### Immunoblot and nuclear/cytosolic extraction

Total protein extracts were lysed in RIPA buffer (Cell Signaling) including 1× cocktails of protease and phosphatase inhibitors (Sigma Aldrich). Gel electrophoresis was performed in a SDS polyacrylamide gel and proteins transferred to a Hybond-P membrane (Millipore). Nuclear and cytosolic fractions were isolated using NE-PER Nuclear and Cytoplasmic Extraction Kit according to the manufacturer's protocol (Thermo Scientific). Binding of each antibody was visualized using the ECL detection system (Thermo Fisher). The images were acquired by myECL Imager (Thermo Fisher) or ChemiDoc XRS (Bio-Rad).

### UAS/Gal4 assay

UAS-GFP x MLL-AF9 cells were transduced with retroviruses MSCV-Gal4 (DNA binding domain, DBD)–NUR77 (ligand binding domain, LBD)–IRES–mCherry. Gal4 is a yeast transcription factor, and the UAS sequence is not recognized by mammalian transcription factors. Fluorescence was detected on ZE5 Cell Analyzer (Biorad).

### Kinase screening

5000 UAS-GFP x MLL-AF9 cells/well were transduced with Gal4-Nur77 and immediately treated with 1 μM concentrations of each compound for 24 h in duplicate. Each 96-multiwell plate contained 16 control wells, and the activity and viability of treated samples were compared with the control cells treated with DMSO. Reporter activity was assessed by mCherry+GFP+ intensity and cell viability assessed by Hoechst 33342 staining. Fluorescence was detected on ZE5 Cell Analyzer (Biorad).

### TurboID sample preparation for MS analysis

For each TurboID-fused construct and drug treatment (MSCV-TurboID and MSCV-TurboID-3xFlag-Gal4 DBD-Nur77 LBD), two biological replicates were performed and analyzed *via* MS. In UAS-GFP x MLL-AF9 cells transduced with MSCV-TurboID-3xFlag-Gal4-Nur77, GFP+ cells were sorted and expanded *in vitro*. MSCV-TurboID was used as negative control. In total, 50 μM of biotin was added to the media for 16 h; 0.2 mM ruxolitinib in DMSO was added 2 h prior biotin, as indicated. Twenty million cells for each experimental point were collected in lysis buffer (25 mM Tris-HCl, 150 mM NaCl, 1% TritonX100, pH 7.2, and protease inhibitor cocktail). The lysate was sonicated at setting 3.5, 8 s × 3. The lysate was incubated on ice for 10 min and spun down 10 min at >13,000 RPM at 4 °C. After lysate was spun down, 20 ml of lysate was aliquoted for Western blot to verify biotinylation/protein expression using streptavidin-HRP antibody (Catalog). To enrich biotinylated proteins from the protein extracts, 80 ml of high-capacity streptavidin agarose resin (Thermo Scientific 20359) was washed twice with lysis buffer, the lysates contained were then incubated with the equilibrated beads on a rotator overnight at 4 °C. The beads were sequentially washed once with 1 ml buffer I (1% SDS in PBS), twice with lysis buffer, and once with washing buffer (50 mM Na2HPO4, 500 mM NaCl, 1% TritonX100, pH 7.4). To completely remove the potential detergent, the beads were in PBS and were resuspended in 300 ml of PBS. The beads were sent immediately on the dry ice for LC-MS/MS analysis. Biotinylated proteins enriched with streptavidin beads were processed into peptides *via* on-bead digestion and analyzed by LC-MS/MS.

### Mass spectrometry data analysis

The streptavidin beads were washed with ammonium bicarbonate and bound proteins were eluted with SDS buffer. Proteins were reduced in dithiothreitol and heated to 95 °C for 10 min. The reduced samples were mixed Tris-Urea buffer and spun in 30,000 MWCO cutoff spin concentrators. Retained proteins were alkylated with Iodoacetamide, then spun and washed to remove unreacted Iodoacetamide. Retained proteins were treated with bicarbonate buffered LysC to relax the protein structure, then digested overnight with sequencing-grade Trypsin. Following digestion, the filters were spun and washed to collect the peptides in the flow through, and remaining detergent was removed using ethyl acetate extraction. Peptides were desalted using porous graphite carbon micro-and the peptides were eluted with acetonitrile in 0.1% TFA and dried, then dissolved in acetonitrile/water in preparation for analysis by Mass spectrometry. The peptides were analyzed using a nano-Elute chromatograph coupled online to a hybrid trapped ion mobility-quadrupole time of flight mass spectrometer (timsTOF Pro, Bruker Daltonics) operated in PASEF mode. Peptides were loaded onto a 75 μm i.d. × 25 cm Aurora Series using constant pressure and eluted over a 2-h gradient. MS1 and MS2 spectra were recorded from m/z 100 to 1700 selecting suitable precursor ions for PASEF-MS/MS in real time from TIMS-MS survey scans, and a polygon filter was applied to the m/z and ion mobility plane to select features most likely representing peptide precursors rather than singly charged background ions. Data from the mass spectrometer were converted to peak lists using Data Analysis and MS2 spectra with charges +2, +3, and +4 were analyzed using Mascot software. Searches were performed against a Mouse UniProt protein database, using trypsin as the digestion enzyme with a maximum of four missed cleavages allowed. The searches were performed with a fragment ion mass tolerance of 50 ppm and a parent ion tolerance of 25 ppm. Carbamidomethylation of cysteine was specified in Mascot as a fixed modification. Deamidation of asparagine, deamidation of glutamine, formation of pyro-glutamic acid from N-terminal glutamine, acetylation of protein N-terminus, and oxidation of methionine were specified as variable modifications. Peptides and proteins were filtered at 1% false discovery rate (FDR) by searching against a reversed protein sequence database (Table S1). The proteomic experiments were performed at the Washington University Proteomics Shared Resource (WU-PSR) (R Reid Townsend MD, PhD, Director, and Robert Sprung, PhD, Co-Director). Isobaric labeling-based relative quantitation was used to score for high-confidence proximity interactors (Table S2).

### Data analysis

Statistical analysis was performed using Prism (Graphpad). *t* test and ANOVA tests were performed, as appropriate. Error bars represent standard deviation. Data points without error bars have standard deviations below Graphpad’s limit to display. Proteomic data were validated using Scaffold 4 (Proteome Software).

## Study approval

All animal procedures were approved by the Institutional Animal Care and Use Committee of Washington University.

## Data availability

All the mass spectrometry data have been deposited in a publicly accessible repository ProteomeXchange Consortium (49) *via* the MassIVE partner repository with the dataset identifier PXD028130 and ftp://massive.ucsd.edu/MSV000088022

## Supporting information

This article contains supporting information.

## Conflict of interest

The authors declare that they have no conflicts of interest with the contents of this article.
