# Supplementary material for: Cytokine exposure mediates transcriptional activation of the orphan nuclear receptor Nur77 in hematopoietic cells
Source: J Biol Chem. 2021 Sep 24;297(5):101240. doi: 10.1016/j.jbc.2021.101240 (PMC8528724; doi:10.1016/j.jbc.2021.101240)
Supplement: Supplemental Figures S1–S6 [file mmc1.pdf]

Supplemental Figure Legends

Supplemental Figure 1. ***Nur77* expression in human and murine samples.**
(A) Expression profile of the 20 highest expressed nuclear receptors (NRs) in 197 human AML cases. NRs are indicated on X-axis. Each dot represents the average expression within an FAB subset of AMLs (M0, M1, M2, M3, M4, M5, M6). (B). Expression profile of Nur77 in sorted normal bone marrow cells. Cell type is indicated on X-axis CD34: CD34+ cells. Pro: promyelocytes. PMNs: neutrophils. CD14: CD14+ cells. CD3: CD3+ cells. CD19: CD19+ cells. (C) Affymetrix expression of Nur77 in tumor samples of acute promyelocytic leukemia *Ctsg-PML-RARA* mice; WT SLAM cells: signaling lymphocyte activation molecule (SLAM) enriched hematopoietic stem cells (HSC); WT KSL stem/progenitor cells: (HSC); WT CMP: common myeloid progenitor; WT MEP: Megakaryocyte/erythrocyte progenitor; WT GMP: granulocyte/macrophage progenitor; WT Pro: promyelocytes; WT PMNs: neutrophils. (D) *Nur77* expression profile in primary murine bone marrow cells transduced with ER-HoxA9. These cells become immortalized and then undergo synchronous neutrophil maturation following estrogen withdrawal. Similar results are observed in additional publicly available datasets with greater granularity of subset flow-based analysis (http://servers.binf.ku.dk/bloodspot/).

Supplemental Figure 2. Analysis of previously reported Nur77-LBD mutants, *in vitro.* (A) UAS-GFP MLL-AF9 leukemia cells were transduced with Gal4-Nur77 or indicated mutations and protein expression was evaluated through Western blot analysis using anti-Flag antibody (M2, Sigma Aldrich). GAPDH was used as a loading control. (B-F) Ratio of GFP+mCherry+ cells relative to total mCherry+ cells in UAS-GFP MLL-AF9 cells transduced with Gal4-Nur77 or indicated mutations and treated as indicated for 24 hours. Each experimental point was performed

in triplicate. * $p < 0.05$. ** $p < 0.01$. *** $p < 0.001$, t-test with Welch's correction. (G) Nuclear and cytosolic protein lysate of UAS-GFP MLL-AF9 cells transduced with MSCV-3xFlag-Gal4-Nur77-IRES-mCherry, treated as indicated for 24 hours, and analyzed by Western blot using anti-Flag antibody. HADC2 and GAPDH were used as a loading control for nuclear and cytosolic extracts, respectively.

Supplemental Figure 3. Analysis of Nur77-LBD cluster mutants, *in vitro*. (A) UAS-GFP MLL-AF9 cells were transduced with Gal4-Nur77 cluster mutations and protein assessed by Western blot using anti-Flag antibody (M2, Sigma Aldrich). GAPDH was used as a loading control. (B) Nuclear and cytosolic protein lysate of UAS-GFP MLL-AF9 cells as indicated and analyzed by Western blot using anti-Flag antibody. HADC2 and GAPDH were used as a loading control for nuclear and cytosolic extracts, respectively (C) UAS-GFP Kit+ cells were transduced with Gal4-Nur77 or cluster mutations, and reporter activity was assessed by GFP. (D-E) UAS-GFP MLL-AF9 leukemia cells were transduced with Gal4-Nur77 or indicated mutations and protein expression was evaluated through Western blot analysis using anti-Flag antibody (M2, Sigma Aldrich). GAPDH was used as a loading control. Each experimental point was performed in triplicate. *** $p < 0.001$, t-test with Welch's correction.

Supplemental Figure 4. Kinase inhibitors library screening. UAS-GFP MLL-AF9 cells were transduced with Gal4-Nur77 and immediately treated with 1 μM concentrations of each compound for 24 hours in duplicate. Cell viability determined by Hoechst 33342 staining. Compounds were organized by pathways: (A) ABL, (B) ALK, (C) ATM, (D) BTK, (E) CDK, (F) EGFR/HER2.

Supplemental Figure 5. Kinase inhibitors library screening. UAS-GFP MLL-AF9 cells were transduced with Gal4-Nur77 and immediately treated with 1 μM concentrations of each compound for 24 hours in duplicate. Cell viability determined by Hoechst 33342 staining. Compounds were

organized by pathways: (A) FAK, (B) IGFR/PDGFR, (C) Kit/Mek, (D) NFkB, (E) TGFb, (F) VEGFR.

Supplemental Figure 6. Proximity labeling of Nur77 in presence vs. absence of Ruxolitinib. (A) UAS-GFP MLL-AF9 cells were transduced as indicated and protein biotinylation was evaluated through Western blot analysis using anti-Streptavidin antibody (Abcam). (B-I). Additional pathway-organized results from TurboID proximity labeling study (Figure 6): (B) Kinases, (C) Phosphatase, (D) Myosin, (E) Ubiquitin, (F) 14-3-3, (G) Chaperone, (H) Ribosome and (I) BTK-SYK2.

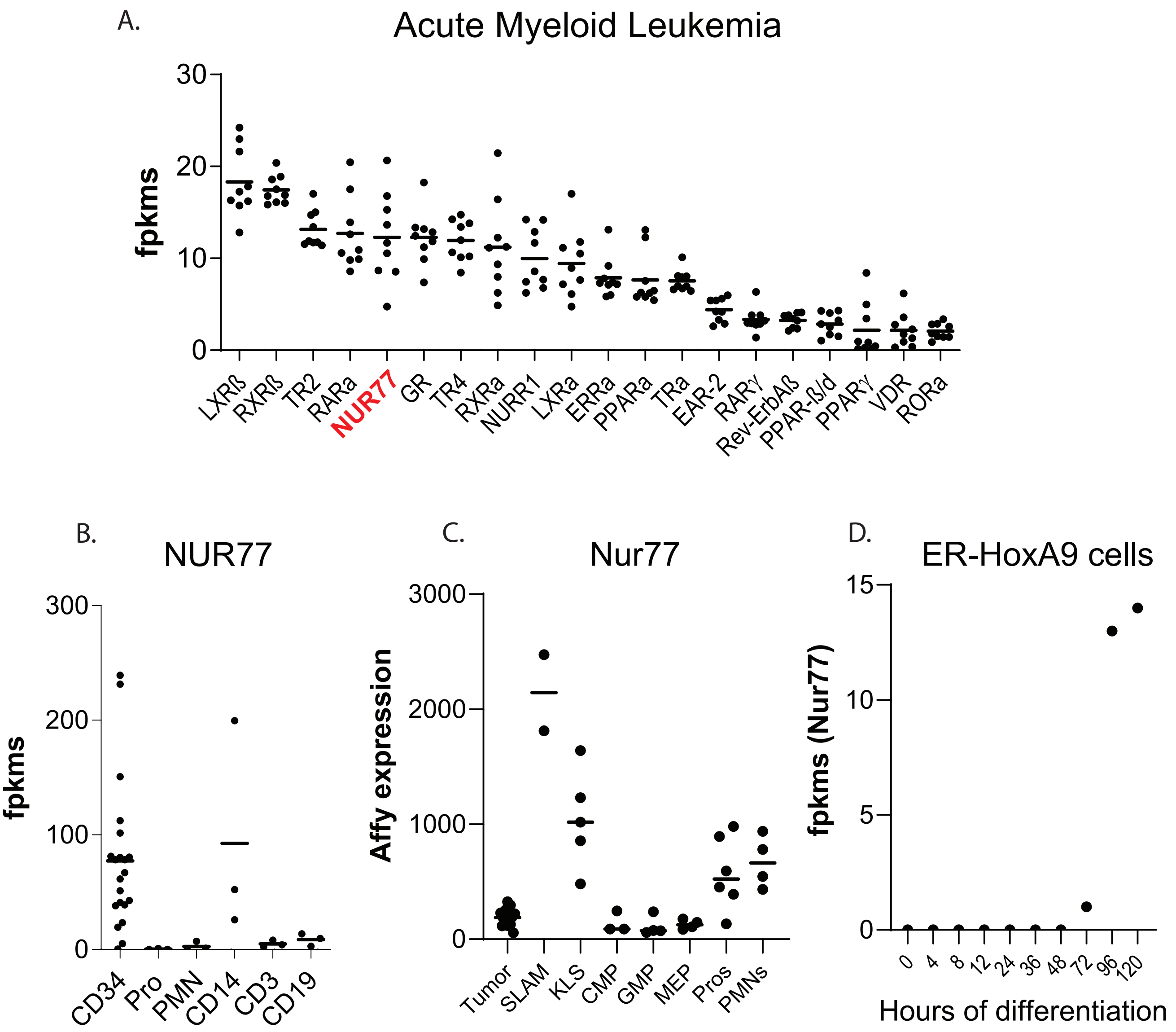

Supplemental Figure 1

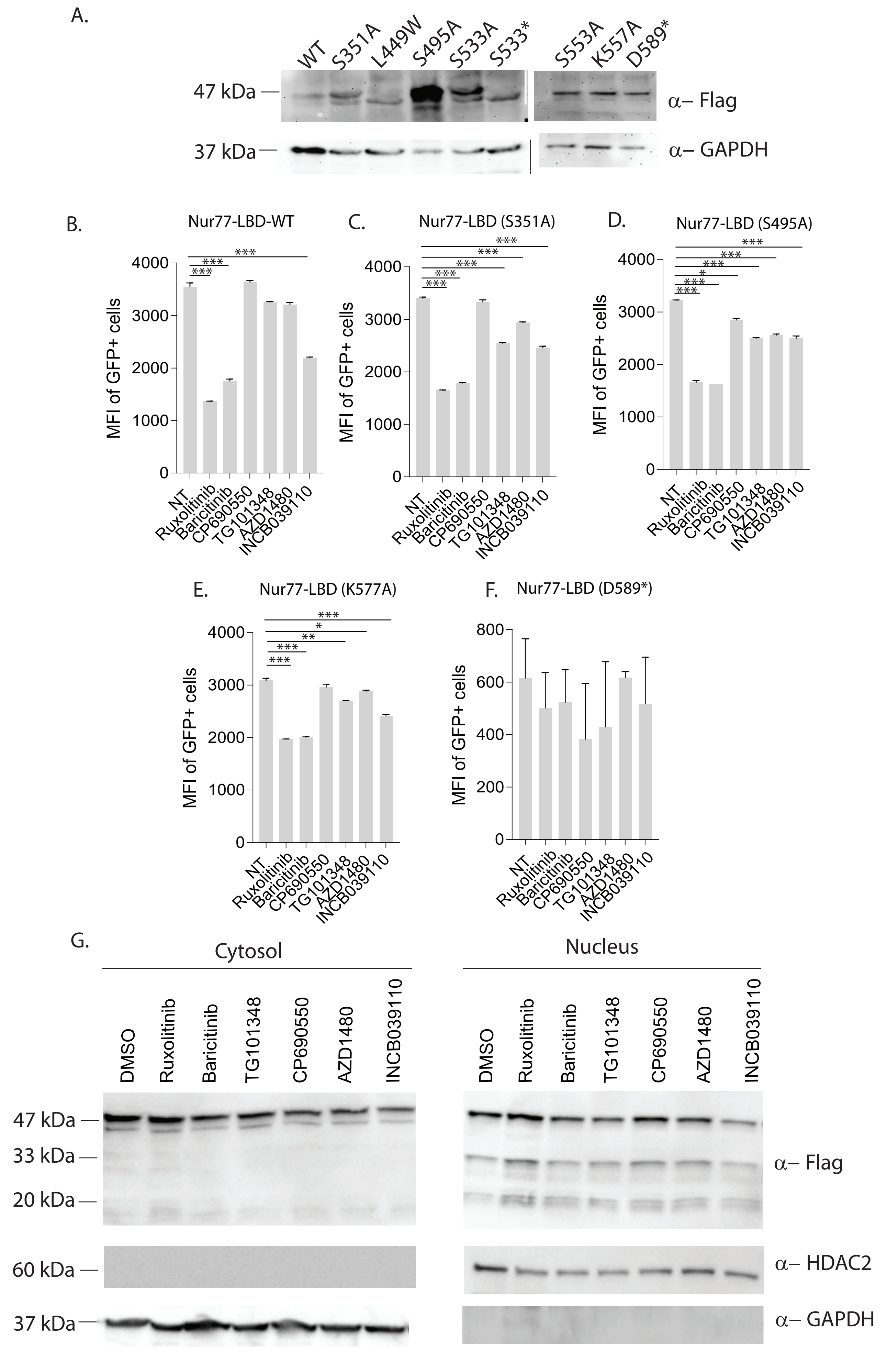

Supplemental Figure 2

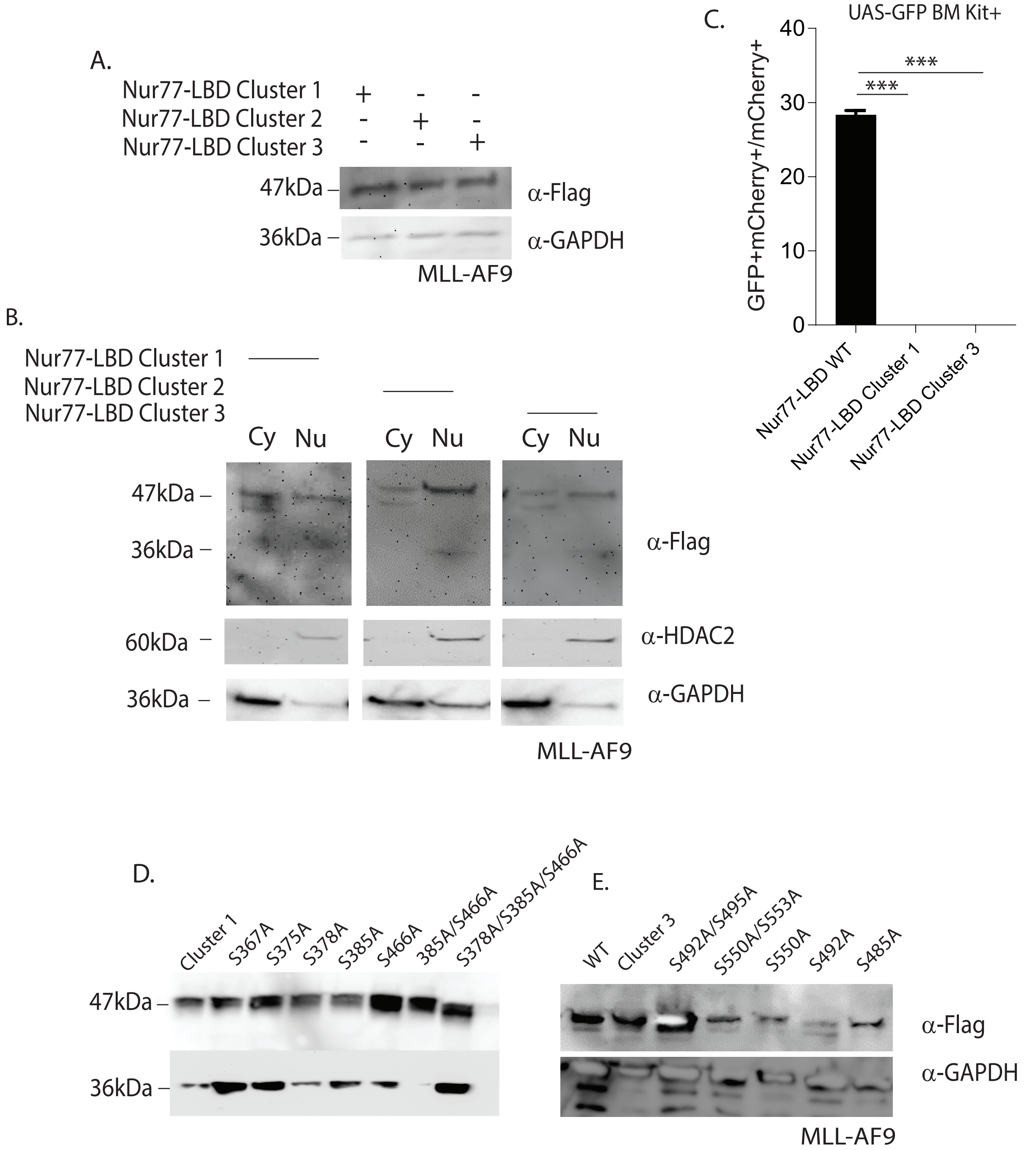

Supplemental Figure 3

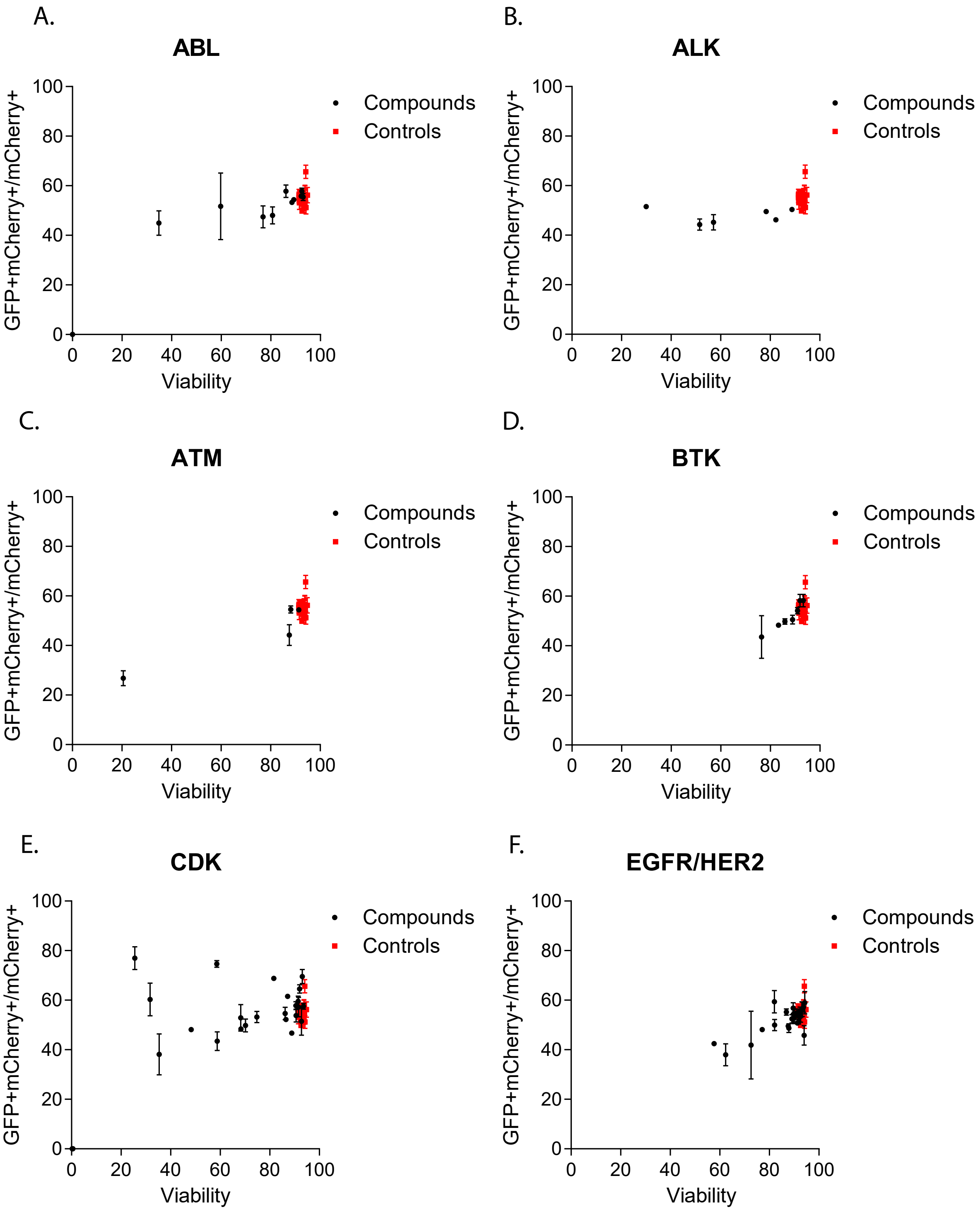

Supplemental Figure 4

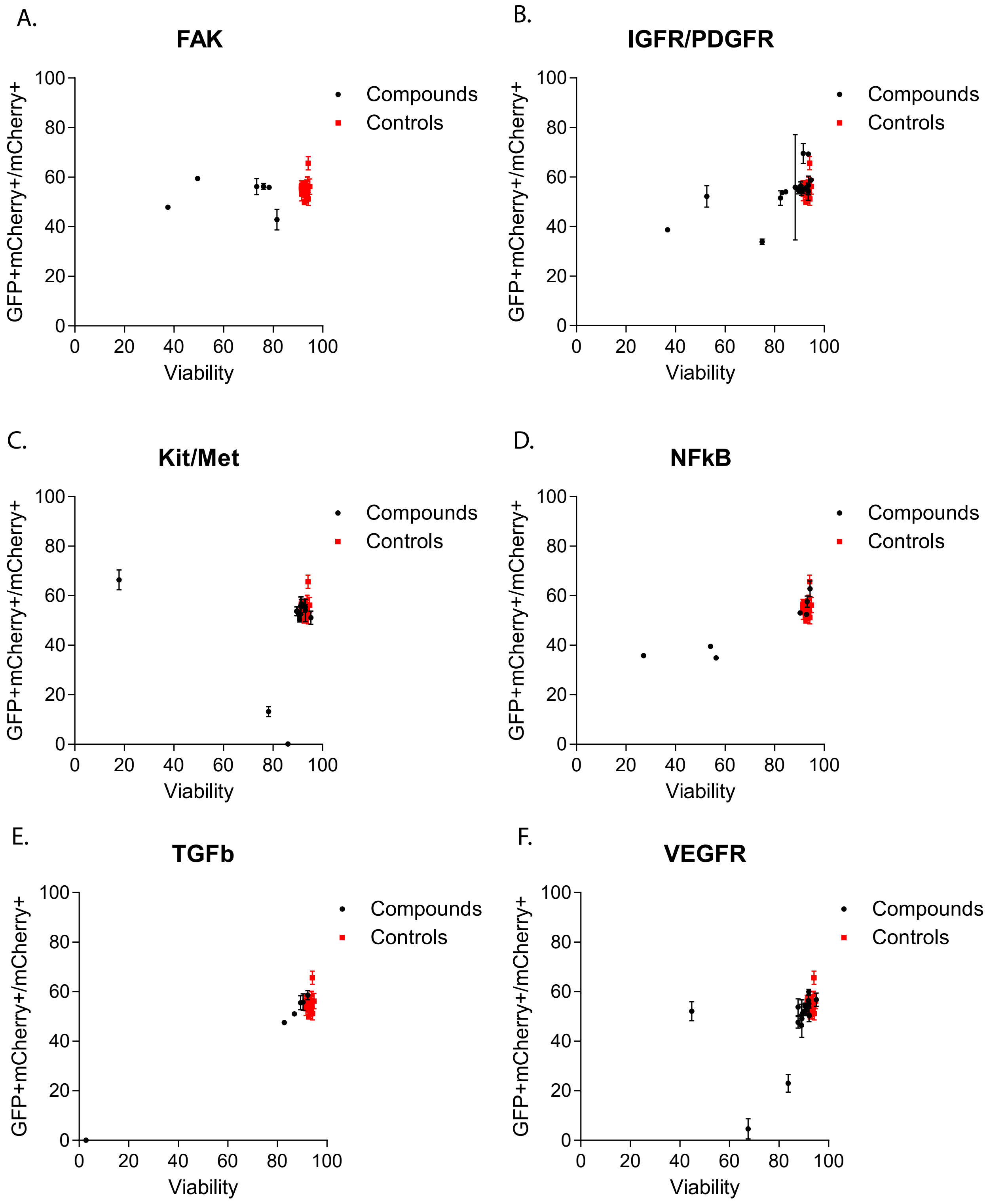

Supplemental Figure 5

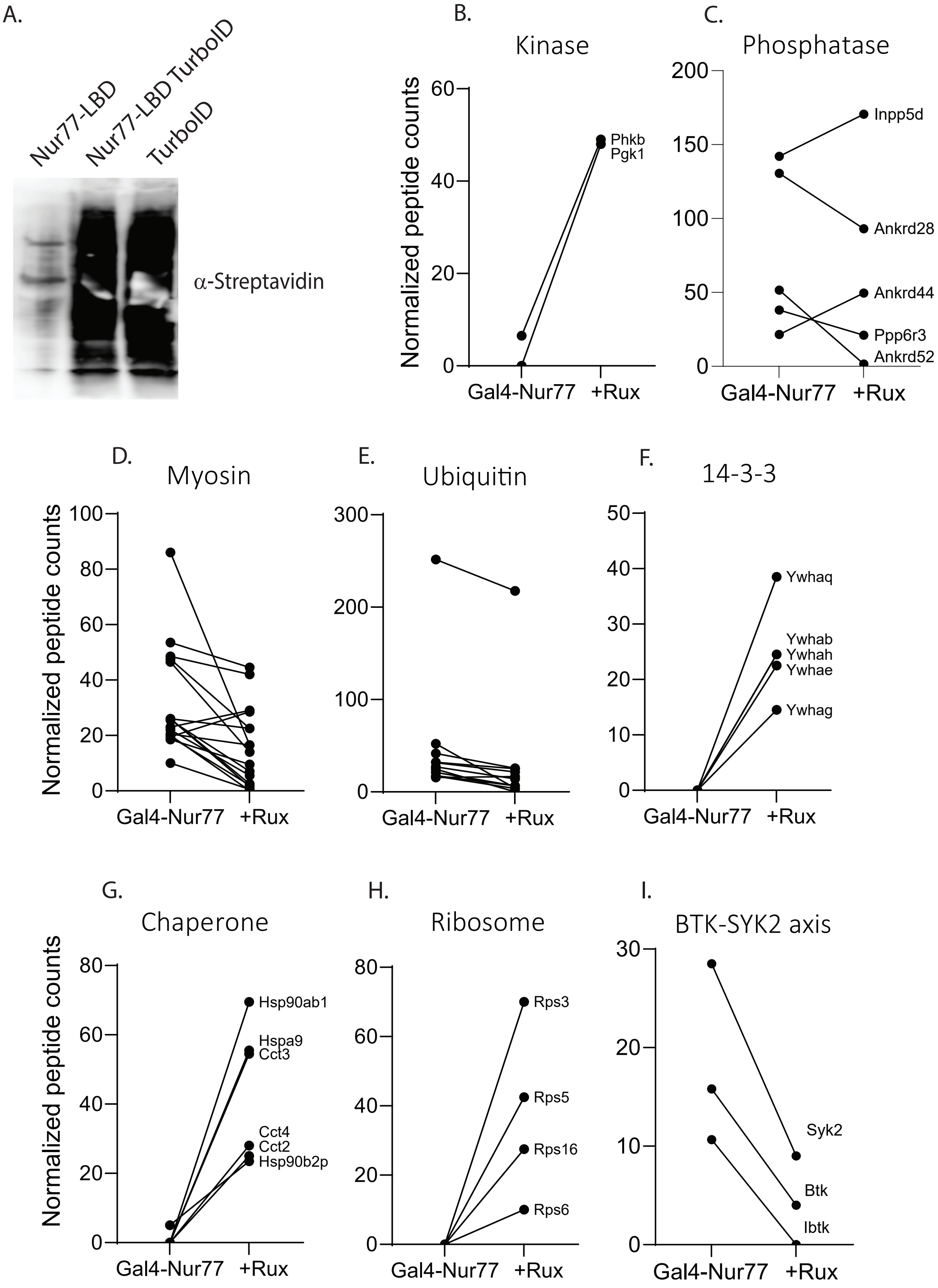

Supplemental Figure 6
